# Stroke Risk Stratification in Incident Atrial Fibrillation: A Sex-Specific Evaluation of CHA2DS2-VA and CHA2DS2-VASc

**DOI:** 10.3390/jcdd12070259

**Published:** 2025-07-05

**Authors:** Jose L. Clua-Espuny, Anna Panisello-Tafalla, Jorgina Lucas-Noll, Eulàlia Muria-Subirats, Teresa Forcadell-Arenas, Juan M. Carrera-Ortiz, Pedro Molto-Balado, Josep Clua-Queralt, Immaculada Fusté-Anguera, Silvia Reverte-Vilarroya

**Affiliations:** 1 Institut Català de la Salut, SAP Terres de l’Ebre, 43500 Tortosa, Spain; 2 Ebrictus Research Group, Research Support Unit Terres de l’Ebre, Institut Universitari d’Investigació en Atenció, Primària Jordi Gol (IDIAP Jordi Gol), 43500 Tortosa, Spain; 3 Primary Health-Care Centre Tortosa Est, Institut Català de la Salut, Primary Care Service (SAP) Terres de l’Ebre, 43500 Tortosa, Spain; jmqcarrera.ebre.ics@gencat.cat (J.M.C.-O.); jcluaq@gmail.com (J.C.-Q.); ifustea.ebre.ics@gencat.cat (I.F.-A.); 4 Direcció Assistencial, Hospital Comarcal Mora d’Ebre, Salut Terres de l’Ebre, 43740 Tarragona, Spain; jlucas.hcme.ste@gencat.cat; 5 Primary Health-Care Centre Amposta, Institut Català de la Salut, Primary Care Service (SAP) Terres de l’Ebre, 43870 Amposta, Spain; eumuria@gmail.com; 6 Primary Health-Care Centre Tortosa Oest, Institut Català de la Salut, Primary Care Service (SAP) Terres de l’Ebre, 43500 Tortosa, Spain; tforcadella.ebre.ics@gencat.cat; 7 CSI Llíria, Departament de Salut de Arnau de Vilanova, Conselleria de Sanitat, Llíria, 46010 Valencia, Spain; pemolto@gmail.com; 8 Nursing Department, Advanced Nursing Research Group at Rovira i Virgili University, Biomedicine Doctoral Programme Campus Terres de l’Ebre, Av. De Remolins, 13, 43500 Tortosa, Spain

**Keywords:** atrial fibrillation, epidemiological studies, CHA2DS2-VASc score, stroke-risk, sex, anticoagulation

## Abstract

(1) Background: In the absence of locally validated tools, the CHA2DS2-VA score has been suggested as a substitute for the CHA2DS2-VASc score. This study compared the potential discrepancies between these scores. (2) Methods: The observational, retrospective, and community-based study included a cohort of 3370 patients with a new diagnosis of atrial fibrillation (AF) between 1 January 2015 and 31 December 2024. (3) Results: AF prevalence was 8.4%, which was significantly higher in men. The mean age was 80.1 (SD ± 6.24) years. Women (42.8%) were older (80.9 SD ± 6.1 vs. 79.5 SD ± 6.23; *p* < 0.001). Men had more instances of diabetes mellitus, peripheral vascular disease, coronary artery disease, and chronic obstructive pulmonary disease, as well as a higher Charlson Comorbidity Index. Conversely, women exhibited a higher proportion ≥75 years, including cognitive impairment, dyslipidemia, and higher stroke risk, as assessed by the CHA2DS2-VASc score (*p* < 0.001) but not by the CHA2DS2-VA score (*p* = 0.071). The CHA2DS2-VA score reduced the sex-based risk stratification differences, and only 3.2% of women were reclassified as being at very low risk (CHA2DS2-VA < 2). (4) Conclusions: The CHA2DS2-VA score notably redefined sex-based thromboembolic risk stratification profiles, with no sex-based disparities in the selection of OAC treatment modality. The clinical utility of CHA2DS2-VA remains a subject of ongoing debate.

## 1. Introduction

Risk stratification for stroke and systemic embolism is essential in the clinical management of patients with atrial fibrillation (AF). Among patients with AF, there is wide variability in the risk of thromboembolic events, regardless of the temporal pattern of AF. Given this substantial risk, oral anticoagulation is advised for all eligible patients, except those at low risk of incident stroke or thromboembolism. Numerous scores for stroke risk stratification in AF have been proposed [1] over the past 15 years, and, in general, these various risk scores showed largely similar discriminative performances. The selection of the CHA2DS2-VASc score as a reference for oral anticoagulant (OAC) prescription guidelines in AF is attributed to its capacity for the accurate identification of patients at genuinely low thromboembolic risk. This discriminatory power in defining a truly low-risk cohort has been a key factor in its adoption by clinical practice guidelines [2]. The high prevalence of AF and the severe consequences of stroke require increased efforts to identify patients with AF, even if they are asymptomatic [3]. There is also a need for user-friendly risk scores to guide appropriate decisions regarding anticoagulation across a wide range of subjects, including more frail, multi-morbid, and clinically complex patients [2,4,5]. A detailed analysis of the components of the CHA2DS2-VASc score identified age and history of prior stroke as the strongest predictors of thromboembolism and stroke in AF patients. However, there has been debate regarding the actual value and significance of the female sex in this context. Recent studies [6,7,8] have highlighted that women with atrial fibrillation face a greater risk of stroke compared to men, and the female sex appears to act more as a ‘risk modifier’ rather than an independent risk factor. This effect is particularly pronounced in older women and when combined with other stroke risk factors. Consequently, these findings hold significant and timely value for supporting the implementation of the CHA2DS2-VASc score in everyday clinical practice.

The 2024 ESC/EACTS Guidelines on AF [9] introduced a significant modification to stroke risk assessment for oral anticoagulation initiation. Specifically, in the absence of locally validated risk stratification tools, the CHA2DS2-VA score is indicated as a replacement for the CHA2DS2-VASc score. This substitution is supported by substantial contemporary evidence indicating the appropriate application of oral anticoagulation using identical treatment thresholds irrespective of patient gender. Consequently, the CHA2DS2-VA score excludes gender as a risk factor based on the premise that female sex does not independently contribute to anticoagulation choices but rather acts as a risk modifier, mainly in older individuals who would already meet criteria for anticoagulation [10]. This adaptation to the CHA2DS2-VA score aims to improve the accuracy of stroke risk assessment and encourage broader adoption of appropriate oral anticoagulation in AF patients, employing uniform treatment cut-offs across genders [11]. This study aims to compare and evaluate potential discrepancies or variations in thromboembolic risk stratification by sex when employing the CHA2DS2-VA score versus the CHA2DS2-VASc score in a contemporary and global cohort of patients with prevalent AF.

## 2. Materials and Methods

### 2.1. Study Design

The “Gender perspective on cardiovascular diseases in the Terres de l’Ebre” (GECA-TE) project is being conducted as a doctoral thesis within the prestigious Biomedicine PhD Program at the Universitat Rovira i Virgili. This doctoral research project aims to comprehensively explore the existence and nature of sex-related differences in various aspects of cardiovascular health, specifically within the Terres de l’Ebre geographical area, and it seeks to contribute to a more nuanced understanding that can inform more effective prevention, diagnosis, and treatment strategies tailored to sex.

This was an observational, retrospective, and community-based study of a cohort of 40,077 of the general population aged 65–90 years between 1 January 2015 and 31 December 2024 without a prior diagnosis of atrial fibrillation or stroke. The protocol received ethics evaluation and approval from the Ethical Committee of Jordi Gol University Institute of Primary Care Research with registration number 24/187-P.

### 2.2. Study Scope

The study was carried out in Terres de l’Ebre (Health Region Terres de l’Ebre, Appendix A), located in the southern part of Catalonia (Spain). It includes 178,112 inhabitants (49.6% women) across 54 municipalities, with an average of 53.8 inhabitants/km^2^ vs. 241.8 inhabitants/km^2^ in Catalonia [12]. It demonstrates the aging of the population, with an aging index (159.5) higher than that of Catalonia (131.3) and Spain (118.4) [13]. This index was obtained through calculating the ratio between the population over 65 years of age and the population under 15 years of age per 100 inhabitants. The population aged 65 years or older represents 31.1% of the overall population. The population in the study has a lower average income than the general population in Catalonia (77.4% vs. 100% per capita) [14,15].

The territory is made up of 4 counties with 11 primary care teams (EAPs), managed by the Catalan Health Institute (ICS), Department of Health (CatSalut). Specialized care is received at the reference hospital located in Tortosa, “Hospital Verge de la Cinta”, which is publicly managed by the ICS. The EAPs (primary care teams) are organized as independent clinical functional teams. The majority of the census population in the territory (98.2%) has an active Shared Health Record of Catalonia (HCC3) available digitally for continuous care monitoring from any center.

### 2.3. Data Collection and Information Sources

The study protocol and previous results have already been detailed in earlier publications [16,17]; therefore, they will not be repeated here. The clinical background data were obtained retrospectively from a computerized database provided to the principal investigator by the Information and Communication Technology Department from the minimum basic dataset at hospital discharge (CMBD-HA) register using the specific International Classification of Diseases (10th version; ICD-10) in a fully encrypted format. The particular data sets utilized for this project were as follows:

1. The HCC3 Patient Episode Dataset for Catalonia (CatSalut, Health Department), which includes demographic and clinical data on all daily inpatient and outpatient admissions in Catalonian hospitals.
2. The 11 EAPs shared a clinical information database for all general practice (E-cap, HCC3) and hospital (E-sap) interactions, including clinical data, symptoms, investigations, diagnoses, comorbidities, prescribed medications, referrals to secondary and tertiary care, and status (alive/dead). Pharmacological variables were collected from the SIRE (Catalan acronym for Integrated Electronic Prescription System).

Data on these factors were collected automatically when possible or manually otherwise.

### 2.4. Study Population

Initially, the study included people ≥65–90 years old, resulting in 40,077 individuals. The primary endpoint of this study was the new diagnosis of atrial fibrillation between 1 January 2015 and 31 December 2024. Secondary outcomes included the evaluation of cognitive impairment, cardiovascular comorbidities, and oral anticoagulation treatment. Consistent with the 2024 ESC/EACTS Guidelines on Atrial Fibrillation, the CHA2DS2-VASc and CHA2DS2-VA scores were calculated for all patients diagnosed with AF within the study period to facilitate comparative analysis. The null hypothesis of this investigation was that there are no statistically significant differences between the two risk scores, nor are there significant differences in these scores when stratified by sex.

### 2.5. Inclusion and Exclusion Criteria

#### 2.5.1. Inclusion Criteria

Patients 65–90 years old, active medical records in any of the health centers with information accessible through the shared history (HCC3), without prior AF or stroke, residence in the territory and assignment to any of the territory’s primary are teams (EAPs). The non-availability or loss of accessibility to the information necessary for the study was considered as a reason for exclusion.

#### 2.5.2. Exclusion Criteria

Previous diagnosis of AF and/or stroke, pacemaker or defibrillator wearer, absence of or lack of access to the individual or their clinical records for any reason, and/or residence outside the Terres de l’Ebre.

### 2.6. Variables

Information regarding atrial fibrillation and relevant cardiovascular comorbidities was systematically collected for each patient. Data acquisition continued until one of the following events occurred: the patient was lost to follow-up, the date of death, or 31 December 2024, whichever came first. The diagnosis of AF strictly adhered to the guidelines set forth by the European Society of Cardiology. Our methodology primarily relied on clinical data recorded within our established AI-driven electronic health record system. Patients were categorized based on whether they had a diagnosis of AF:

- For individuals diagnosed with AF during the follow-up period, relevant data were extracted at the time of their AF diagnosis and continued to be collected until the end of their individual follow-up period.
- For patients who did not develop AF during the entire follow-up, their data were gathered as a mean value recorded throughout their observation period.
- For cardiovascular risk factors and diagnostics using specific International Classification of Diseases (ICD–10) code prefixes for cerebrovascular disease (ischemic stroke or transient ischemic attack, I63, G45), heart failure (I50-51), ischemic heart disease (stable or unstable angina, percutaneous coronary intervention, coronary artery bypass grafting, or myocardial infarction) (I20-I25), hypertension (I10–I15), hypercholesterolemia (E78), diabetes mellitus (E10–E14), body mass index (BMI), chronic kidney disease (CKD) (N18), and estimated glomerular filtration rate (eGFR ml/min/1.73 m^2^).

The incidence of stroke/thromboembolism during follow-up was ascertained employing International Classification of Diseases codes extracted from the electronic health records of all patients within the study cohort. Specifically, we utilized ICD-10 codes for ischemic stroke (I63.x), transient ischemic attack (G45.x), and systemic embolism (I74.x for arterial embolism/thrombosis). To ensure that only incident events occurring subsequent to the date of atrial fibrillation (AF) diagnosis were considered as study outcomes, the following rigorous methodology was implemented:

(a) Event Date Filtering: Each identified stroke or thromboembolic event record was time-stamped, and any event occurring on or prior to the date of AF diagnosis was systematically excluded from the outcome analysis.
(b) Exclusion of Pre-existing Stroke: Furthermore, patients with a documented history of stroke/thromboembolism preceding their incident AF diagnosis were excluded from study enrolment.

1/ Clinical scores: Charlson Comorbidity Index to assess a patient’s comorbidity burden, CHA2DS2-VASc and CHA2DS2-VA, and Pfeiffer Short Mental Status Questionnaire score. The annual stroke risk estimation was calculated according to CHA2DS2-VASc and CHA2DS2-VA scores [18]. The term “sex” has been used to refer to the biological and physical attributes as recorded in patient databases.
2/ Antiplatelet and/or oral anticoagulation treatment.
3/ Vital status (dead/alive) at the end of the study. All participants were followed from 1 January 2015 until 31 December 2024, loss to follow-up, or date of death, whichever occurred first.

### 2.7. Statistical Analysis

The characteristics of the population were defined through a descriptive statistical analysis. Baseline characteristics are presented as counts and percentages, mean and standard deviation (SD) for normally distributed continuous variables, or median for non-normally distributed continuous variables, as appropriate. Quantitative variables were examined with Student’s t-distribution for independent samples, while qualitative variables were analyzed with the chi-square distribution according to bivariate analysis for normal distributions.

The stroke incidence density/1000 people/year and the registered prevalence of cognitive decline were calculated for each group. The incidence rate was calculated using person-years as the denominator. This denominator represented the total person-time at risk, computed as the sum of the follow-up duration for each individual from the date of incident atrial fibrillation diagnosis until the end of the study period or the date of the exclusion event, whichever occurred first. A two-sided *p*-value < 0.05 was considered statistically significant. All statistical analyses were conducted using IBM SPSS Statistics version 21.0.

## 3. Results

Table 1 presents the baseline characteristics of 40,077 individuals aged 65–90 years enrolled in a study investigating new-onset atrial fibrillation. The mean age of the study population was 77.4 years (SD ± 6.60). Women constituted 51.3% and were significantly older compared to men (77.6 SD ± 6.63 vs. 77.2 SD ± 6.56; *p* < 0.001). Significant sex-based differences were observed in the prevalence of cardiovascular diseases and risk factors. Men presented with a higher prevalence of atrial fibrillation, heart failure, diabetes mellitus, stroke, peripheral vascular disease, coronary artery disease, chronic kidney disease, and chronic obstructive pulmonary disease (COPD), and a higher risk of stroke as measured by the CHA2DS2-VA score (*p* < 0.001). Conversely, women had a higher prevalence of cognitive impairment and dyslipidemia; a higher risk of stroke as measured by the CHA2DS2-VASc score (*p* < 0.001); received less anticoagulant treatment; had fewer average hospital visits; and experienced higher mortality.

Table 2 presents the baseline characteristics of a cohort of 3370 patients who received a diagnosis of atrial fibrillation during the study’s period. The mean follow-up duration for this cohort was 26.16 (SD ± 20) months. The mean age of the study cohort was 80.1 years (SD ± 6.24). Women constituted 42.7% and were significantly older compared to men (80.9 SD ± 6.1 vs. 79.5 SD ± 6.23; *p* < 0.001). Significant sex-based differences were observed in the prevalence of cardiovascular risk factors. Men presented with a higher prevalence of age 65 to 74 years, diabetes mellitus, vascular peripheral disease, coronary artery disease, and chronic obstructive pulmonary disease; and a higher predicted mortality rate as measured by the Charlson index. Conversely, women had a higher prevalence of age ≥75 years, cognitive impairment, and dyslipidemia; a higher risk of stroke as measured by the CHA2DS2-VASc score (*p* < 0.001), but not if measured by CHA2DS2-VA (*p* = 0.071).

Table 3 shows how suppressing the sex variable leads to a redistribution of women into a lower risk stratification, thereby aligning it with the proportion of the population in each risk stratum as determined by the CHA_2_DS_2_-VASc for men. This equalizes the population risk distribution by removing an overestimation of risk in women, notably in the low-risk segment, thus classifying the population into a lower-risk stratum. Consequently, 46 women (3.2% of the female cohort) were reclassified to a low-risk category (CHA2DS2-VA < 2), suggesting that oral anticoagulation initiation could be reconsidered or withheld for these individuals, in alignment with the 2024 ESC/EACTS guidelines. Using the CHA2DS2VASc, the proportion of men with a score < 4 is significantly higher, while above a score of 5, the proportion of women is significantly higher. In contrast, through the use of CHA2DS2VA, 46 women (3.2%) are identified with a score < 2, with the differences disappearing in the remaining strata except in score 5, where the proportion of men is significantly higher.

Table 4 shows the incidence rate per 100 person-years by CHA2DS2VASc vs. CHA2DS2VA according to the registered strokes in the study period. The follow-up time was counted from the date on which atrial fibrillation was diagnosed until the end of the study or withdrawal from it due to the patient’s death. The mean follow-up time was 4.96 years (SD ± 1.17). No significant differences between the two groups were suggested by the rate ratio for individuals with a score of *≤6*. By decreasing the proportion included in the high strata, there is an increase in the incidence density above the score ≥5 with the CHA2DS2VA scale.

## 4. Discussion

In the cohort of 3370 patients newly diagnosed with atrial fibrillation, the main findings were: (1) an average age of 80.1 years, notably higher than in many other atrial fibrillation studies [19,20]; (2) an 8.4% prevalence of AF, which was significantly higher in men; and (3) comparable performance of the CHA_2_DS_2_-VA and CHA_2_DS_2_-VASc scores in predicting ischemic stroke. The application of the CHA2DS2-VA score led to a substantial reduction in the sex-based profile differences in thromboembolic risk stratification observed with the CHA2DS2-VASc score. Specifically, only a small fraction of women (3.2%) was consequently reclassified to a low-risk category (CHA2DS2-VA = 1). For these individuals, the initiation of OAC could be reconsidered or potentially withheld, guided by a patient-centered and shared decision-making approach. It is important to note that this observed effect does not imply a superior predictive performance of CHA2DS2-VA’s in stratifying stroke risk. Instead, the study aimed to provide empirical evidence on the consequences of applying this new scoring system within a real-world, elderly cohort, particularly focusing on how it redefines sex-based risk stratification and its practical implications for OAC decisions.

The disparities in the prevalence of cardiovascular diseases or risk factors between women and men were more noteworthy in the general population (Table 1) compared to those observed within the atrial fibrillation population (Table 2). This suggests that the previously reported sex-based differences in thromboembolic risk may no longer be evident in contemporary cohorts of patients with AF. The development of AF, particularly in an elderly, multimorbid cohort, often implies a high cumulative burden of cardiovascular risk factors for both sexes. When both men and women within an AF cohort have reached a similar high burden of established risk factors, the independent contribution of biological sex to additional thromboembolic risk, beyond these highly prevalent comorbidities, may become less discriminatory or even statistically non-significant. In other words, the presence of AF itself, coupled with the high comorbidity burden common in older AF patients, might overshadow or homogenize some of the baseline sex-based risk factor differences, leading to a diminished independent role of sex in predicting future thromboembolic events. This perspective aligns with the evolving understanding that, while sex may modify risk, its independent contribution to thromboembolic risk in the context of other potent risk factors (like those in CHA2DS2-VA) may become less pronounced.

The finding that women in this study were significantly older (average 80.9 years) compared to men (average 79.5 years) aligns with several studies suggesting that women tend to develop atrial fibrillation around 5 to 10 years later than men on average. In studies on very elderly populations, the trend often shifts towards a higher proportion of women [21], in contrast with the male predominance often observed in younger atrial fibrillation populations, underscoring the importance of considering age when analyzing gender differences in this condition.

Age is a significant predictor of various health events and responses to treatment. The high average age of the current cohort suggests that its findings will be particularly relevant to understanding and managing atrial fibrillation in the very elderly, a group that may have different disease characteristics and treatment responses compared to younger elderly individuals. Atrial fibrillation in the very elderly can frequently be asymptomatic or present with non-specific symptoms, potentially leading to underdiagnosis and delayed treatment, which could increase the risk of adverse outcomes. In this advanced age group, patients are more likely to have multiple comorbidities such as high blood pressure, coronary artery disease, heart failure, obesity, and chronic kidney disease, which can contribute to the development and progression of atrial fibrillation and have a negative impact on survival [22]. The risk of serious complications, particularly stroke and systemic embolism, is substantially elevated in very elderly individuals with atrial fibrillation, making the decision regarding anticoagulation therapy a critical aspect of management. This decision requires a careful balance between the risk of thromboembolism and the increased risk of bleeding often associated with anticoagulants in older adults, especially considering potential frailty and cognitive impairment. Rhythm control strategies, which aim to restore and maintain a normal heart rhythm, might be less effective in this age group and could carry a higher risk of adverse effects from antiarrhythmic medications. Non-pharmacological interventions like catheter ablation might be considered in carefully selected patients. The presence of frailty and cognitive decline can further complicate management by affecting medication adherence and the ability of patients to participate in treatment decisions. The growing prevalence of multimorbidity and AF significantly burdens global healthcare systems. In addition, three-quarters of atrial fibrillation patients take at least five medications. To develop effective strategies, improve patient outcomes, and address the burden of AF, it is essential to understand the relationship between these conditions.

Our study included the comorbidities of the CHA2DS2-VASc scale and not all those defined by EHRA-PATHS [19] as relevant in patients with AF. Based on the prevalence of specific comorbidities, the modification of the risk calculation from the CHA2DS2-VASc to the CHA2DS2-VA scale resulted in a one-point decrease (sex-woman variable: 1) in the women’s group. The CHA2DS2-VA score exhibited comparable predictive accuracy for thromboembolic events to the established CHA2DS2-VASc score, and only a small fraction of women (3.2%) were re-categorized as being at a low risk of stroke, suggesting that OAC initiation should be reconsidered or withheld for these individuals. Given their comparable performance, the non-sex-based CHA2DS2-VA score may simplify the initial decision-making process for initiating OAC in patients with AF and would be inclusive of individuals who are “non-binary, transgender, or are undergoing sex hormone therapy [21].

Moreover, it is unclear whether this decrease in thromboembolic risk is associated with a decrease in overall cardiovascular risk related to the comorbidities, since the CHA2DS2-VA scale is characterized by accounting for risk factors in a binary manner and the cumulative number of diseases rather than the overall underlying complexity of multimorbidity [23,24]. Due to significant advantages in big data processing, artificial intelligence is increasingly being integrated into risk stratification and clinical decision support systems for atrial fibrillation (AI) patients. Notably, models employing AI have demonstrated enhanced performance in predicting stroke risk when compared to the conventional CHA2DS2-VASc scoring system [25,26].

Regarding anticoagulant prescription patterns, no statistically significant sex-based disparities were evident in the selection of OAC treatment modality, but a notable prevalence of subtherapeutic dosing, significantly higher in female patients, has been reported [27,28]. If the Sc (sex category) component is removed from the CHA_2_DS_2_-VASc score, it could contribute to the established pattern of suboptimal anticoagulation in women with AF [29]. On the other hand, while vitamin K antagonists constitute 32% of OAC prescriptions and necessitate regular monitoring via the international normalized ratio, the lack of an equivalent objective adherence monitoring mechanism for NOACs remains a significant clinical challenge [30] in the context of adherence to oral anticoagulants among patients with atrial fibrillation. Ultimately, the interaction between female sex, comorbidities, oral anticoagulant use, and stroke risk may vary across the age spectrum.

Finally, as strengths, the study was based on a multicenter, community-based cohort and capable of contributing valuable data to the existing body of knowledge on atrial fibrillation in a large patient group. Our findings provide epidemiological data for comparing the predictive performance of CHA2DS2-VA and CHA2DS2-VASc scores in stratifying stroke risk in AF patients. In addition, several limitations deserve consideration. The inclusion of patients with incident atrial fibrillation introduces a potential for survivorship bias. Specifically, individuals with a history of stroke prior to AF diagnosis or those with long-standing, established AF may represent distinct phenotypes with different disease trajectories, comorbidity profiles, and treatment histories compared to those with newly diagnosed AF. Consequently, the observed associations and predictive performances of the CHA2DS2-VA and CHA2DS2-VASc scores may not be directly translatable to cohorts comprising patients with chronic, established AF or those who have survived a stroke. Some scores might be more sensitive or specific in identifying risk in a primary prevention cohort (patients without prior stroke) compared to a secondary prevention cohort (patients with prior stroke). Furthermore, as the study cohort exclusively comprised patients aged >65 years, all individuals inherently accrued at least one point from the age component (either ‘Age 65–74 years’ or ‘Age ≥ 75 years’) in both the CHA2DS2-VA and CHA2DS2-VASc scores. This means that it lacks patients in the ‘truly very low risk’ category (CHA2DS2-VA score = 0), thereby precluding the provision of insights into risk stratification for younger AF patients.

Future research directions include investigating the intricate relationships between specific comorbidities and the pathogenesis of atrial fibrillation and analyzing age- and sex-stratified treatment modalities and their associated outcomes. Furthermore, prospective studies are warranted to validate these findings across the entire continuum of AF patients and ascertain whether the CHA2DS2-VA score will lead to better implementation of oral anticoagulation across the heterogeneous spectrum of at-risk atrial fibrillation patients, excluding only those at “truly very low risk” of thromboembolism, and to confirm that this occurs equitably, aligning with the objective of ensuring universal access to effective and appropriate care.

## 5. Conclusions

1/ The application of the CHA2DS2-VA score notably redefined sex-based thromboembolic risk stratification profiles compared to the CHA2DS2-VASc score, leading to a substantial reduction in previously observed sex-based disparities in risk categories within this elderly cohort.
2/ The 3.2% of women were reclassified to a low-risk category (CHA2DS2-VA < 2), suggesting that oral anticoagulation could be reconsidered or withheld for these individuals, guided by a patient-centered approach.
3/ No statistically significant sex-based disparities were evident in the selection of OAC treatment modality.
4/ While this study provides empirical evidence on the practical consequences of applying the CHA2DS2-VA score in a real-world, elderly cohort, the broader clinical utility of adopting this score for comprehensive stroke risk stratification across diverse AF populations remains a subject of ongoing debate and warrants further prospective research.

## Figures and Tables

**Table 1:** Baseline characteristics of cases by sex. General population.

Variables	Men	(%)	Women	(%)	*p*	All (%)
All (n%)	19,531	48.7%	20,548	51.3%	-	40,077
AF	1928	9.9%	1442	7.0%	<0.001	3370 (8.4%)
Age average	77.28 ± 6.56		77.6 ± 6.63		<0.001	77.4 ± 6.60
CHA_2_DS_2_-VASc	2.7 ± 1.1		3.6 ± 1.1		<0.001	3.2 ± 1.2
CHA2DS2-VA	2.8 ± 1.1		2.6 ± 1.1		<0.001	2.68 ± 1.1
Heart failure	1790	9.2%	1558	7.6%	<0.001	3348 (8.4%)
Hypertension arterial	11,526	59.0%	12,218	59.5%	0.360	23,744 (59.2%)
Age 65 to 74 years	7748	39.6%	7765	37.8%	<0.001	15,513 (38.7%)
Age ≥ 75 years	11,783	60.3%	12,783	62.6%	<0.001	24,566 (61.3%)
Diabetes mellitus	5763	29.5%	4498	21.9%	<0.001	10,261 (25.6%)
Stroke/TIA/Systemic embolism	843	4.7%	724	3.5%	<0.001	1567 (3.9%)
Vascular peripheral disease	1983	10.2%	798	3.9%	<0.001	2781 (6.9%)
Ischemic cardiomyopathy	2073	10.6%	917	4.5%	<0.001	2990 (7.5%)
BMI ^1^ (kg/m^2^)	28.1 ± 4.5		28.4 ± 5.7		<0.001	28.3 ± 5.2
Charlson index	1.5 ± 1.4		1.2 ± 1.2		<0.001	1.38 ± 1.9
Dementia/cognitive impairment	1452	7.4%	2225	10.8%	<0.001	3677 (9.2%)
Pfeiffer score	2.72 ± 3.20		3.63 ± 3.35		<0.001	3.23 ± 3.3
Chronic Kidney Disease	3181	16.3%	3080	15.0%	<0.001	6261 (15.6%)
Glomerular filtration rate (ml/min/1.73 m^2^)	72.2 ± 18.0		73.4 ± 17.6		<0.001	72.9 ± 17.7
COPD ^2^/asthma/bronchitis	2895	14,8%	2224	10.8%	<0.001	5119 (12.8%)
OSAHS ^3^	966	4.9%	473	2.3%	<0.001	1439 (3.6%)
Dyslipidemia	8394	43.0%	10,623	51.7%	<0.001	19,017 (47.5%)
Statins	6006	30.8%	6326	30.8%	0.940	12,332 (30.8%)
Antiaggregants	3411	17.5%	2335	11.4%	<0.001	5746 (14.3%)
Anticoagulation	1977	10.1%	1468	7.1%	<0.001	3445 (8.6%)
Hospital visits	0.36 ± 1.3		0.27 ± 0.95		<0.001	0.31 ± 1.15
Active medications	5.17 ± 4.3		5.76 ± 4.40		<0.001	5.48 ± 4.36
Exitus	14,492	74.2%	17,173	83.6%	<0.001	31,665 (79.0%)

^1^ BMI: Body Mass Index; ^2^ COPD: Chronic Obstructive Pulmonary Disease; ^3^ OSAHS: Obstructive Sleep Apnea/Hypopnea Syndrome.

**Table 2:** Baseline characteristics of cases by sex. Population with new AF.

Variables	Men	(%)	Women	(%)	*p*	All (%)
All (n%)	1928	57.2%	1442	42.8%	<0.001	3370
Age average	79.5 ± 6.23		80.9 ± 6.1		<0.001	80.1 ± 6.24
CHA_2_DS_2_-VASc	3.58 ± 1.18		4.51 ± 1.12		<0.001	3.98 ± 1.24
CHA2DS2-VA	3.58 ± 1.18		3.51 ± 1.12		0.071	3.55 ± 1.16
Heart failure	690	35.8%	543	37.7%	0.278	1233 (36.6%)
Hypertension arterial	1439	74.6%	1091	75.7%	0.520	2530 (75.1%)
Age 65 to 74 years	460	23.8%	246	17.1%	<0.001	706 (20.94%)
Age ≥ 75 years	1468	76.1%	1196	82.9%	<0.001	2664 (79.1%)
Diabetes mellitus	727	37.7%	461	32.0%	0.001	1188 (35.3%)
Stroke/TIA/Systemic embolism	194	10.1%	137	9.5%	0.599	331 (9.8%)
Vascular peripheral disease	351	18.2%	119	8.3%	<0.001	470 (13.8%)
Ischemic cardiomyopathy	375	19.5%	162	11.2%	<0.001	537 (15.8%)
BMI ^1^ (kg/m^2^)	29.07 ± 5.1		28.53 ± 6.2		0.022	29.2 ± 5.5
Charlson index	2.27 ± 1.5		1.91 ± 1.38		<0.001	2.10 ± 1.45
Dementia/cognitive impairment	196	10.2%	212	14.7%	<0.001	408 (12.1%)
Pfeiffer score	2.14 ± 2.7		3.31 ± 3.0		<0.001	2.71 ± 2.9
Chronic Kidney Disease	581	30.1%	417	28.9%	0.446	998 (29.6%)
Glomerular filtration rate (ml/min/1.73 m^2^)	65.5 ± 20.0		64.7 ± 19.8		0.356	65.16 ± 19.9
COPD ^3^/asthma/bronchitis	454	23.5%	223	15.5%	<0.001	677 (20.1%)
OSAHS ^2^	170	8.8%	54	3.7%	<0.001	224 (6.6%)
Dyslipidemia	933	48.4%	774	53.7%	0.002	1707 (50.7%)
Statins	721	37.4%	505	35.0%	0.158	1226 (36.4%)
Antiaggregants	122	6.3%	48	3.3%	<0.001	170 (5.0%)
Anticoagulation	1522	78.9%	1140	79.0%	0.9694	2662 (78.9%)
VKAs ^4^	492	32.3%	378	33.1%	0.6810	870 (32.6%)
NOACs ^5^	1030	67.6%	762	66.8%	0.6810	1792 (67.3%)
Hospital visits	0.68 ± 1.7		0.58 ± 1.51		0.070	0.64 ± 1.64
Active medications	8.03 ± 4.6		8.57 ± 4.7		0.001	8.26 ± 4.68
Exitus	1445	74.9%	1125	78.0%	0.095	2570 (76.3%)

^1^ BMI: Body Mass Index; ^2^ COPD: Chronic Obstructive Pulmonary Disease; ^3^ OSAHS: Obstructive Sleep Apnea/Hypopnea Syndrome; ^4^ VKAs: vitamin K antagonists; ^5^ NOACs: Non-vitamin K oral anticoagulants.

**Table 3:** Thrombotic Risk Stratification by CHA_2_DS_2_-VASc and CHA_2_DS_2_-VA in Men and Women with New AF.

Tab	CHA2DS2VASc			CHA2DS2VA			
Score	Women N1 (%)	Men n1 (%)	*p*	Women N2 (%)	Men n2 (%)	*p*	Total Registered Stroke
1	-	61 (3.1%)	<0.001	46 (3.2%)	61 (3.1%)	0.9549	-
2	46 (3.2%)	276 (14.3%)	<0.001	210 (14.5%)	276 (14.3%)	0.8784	10 (2.99%)
3	210 (14.6%)	593 (30.7%)	<0.001	459 (31.8%)	593 (30.8%)	0.5301	49 (14.67%)
4	459 (31.8%)	589 (30.5%)	0.4489	481 (33.3%)	589 (30.5%)	0.0902	112 (33.53%)
5	481 (33.5%)	307 (15.9%)	<0.001	183 (12.7%)	307 (15.9%)	0.0098	86 (25.74%)
6	183 (12.6%)	88 (4.5%)	<0.001	58 (4.0%)	88 (4.5%)	0.4969	58 (17.36%)
7	58 (4.0%)	14 (0.7%)	<0.001	5 (0.3%)	14 (0.7%)	0.2214	19 (5.68%)
8	5 (0.3%)	-	0.0327	-	-		-
9	-	-		-	-		-
Total	1442 (7.0%)	1928 (9.9%)	<0.001	1442 (7.0%)	1928 (9.9%)	<0.001	334

N1: Number of women; n1: number of men; N2 number of women; n2: number of men.

**Table 4:** Stroke Incidence Rates per 100 Person-Years: Comparison of CHA2DS2VASc and CHA2DS2-VA Scores.

	CHA2DS2VASc			CHA2DS2-VA			
Score	AF (N)	Stroke (n)	Incidence Rate Per 100 Person-Years CI95%	AF (N)	Stroke (n)	Incidence Rate Per 100 Person-Years CI95%	Rate Ratio CI95%
1	61	-		107	-	-	
2	322	7	0.45 [0.18–0.90]	486	10	0.41 [0.20–0.76]	1.08 [0.41–2.84]
3	803	32	0.81 [0.56–1.15]	1052	49	0.94 [0.70–1.25]	0.85 [0.55–1.34]
4	1048	77	1.48 [1.17–1.85]	1070	112	2.11 [1.74–2.54]	0.70 [0.52–0.93]
5	788	112	2.82 [2.35–3.43]	490	86	3.50 [2.80–4.33]	0.81 [0.61–1.07]
6	271	60	4.42 [3.37–5.69]	146	58	7.75 [5.89–10.02]	0.56 [0.39–0.81]
7	72	41	10.93 [7.85–14.83]	19	19	19.00 [11.44–29.67]	0.56 [0.33–0.98]
8	5	5	-	--	-		
9	-	-	-	-			
Total	3370	334	2.00 [1.79–2.22]	3370	334	2.00 [1.79–2.22]	

**Figure A1:**
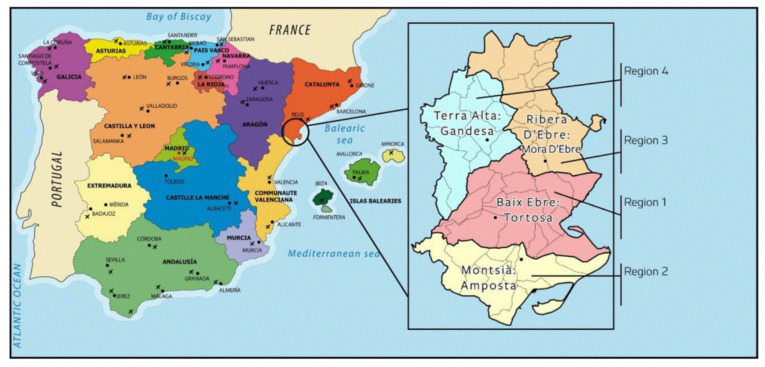
Regional map of the Terres de l’Ebre study area within Spain.

## Data Availability

The data supporting the findings of this study are not currently publicly available but can be requested from the authors upon reasonable request. These data will be available through an institutional repository following the public defense of the corresponding PhD thesis.

## Abbreviations

The following abbreviations are used in this manuscript:

AF	Atrial Fibrillation
OAC	Oral Anticoagulants
ESC-EACTS Guidelines	Guidelines developed by the European Society of Cardiology (ESC) and the European Association for Cardio-Thoracic Surgery (EACTS)
GECATE	Acronym for “Gender perspective on cardiovascular diseases in the Terres de l’Ebre”
ICS	Acronym for “Catalan Health Institute”
EAPs	Acronym for “Primary Care teams”
HCC3	Acronym for “Shared clinical record of Catalonia”
SIRE	Acronym for “Integrated Electronic Prescription System”
COPD	Chronic Obstructive Pulmonary Disease
BMI	Body Mass Index
OSAHS	Obstructive Sleep Apnea/Hypopnea Syndrome
VKAs	Vitamin K antagonists
NOACs	Non-vitamin K oral anticoagulants
